# Meeting of Fulton County Medical Society

**Published:** 1914-08

**Authors:** R. R. Daly


### FULTON COUNTY MEDICAL SOCIETY

### Regular Meeting Thursday, August 6, 1914.

By R. R. Daly, M. D.

Dr. J. S. Derr showed skiagrams of Spinal Abcesses. They are so far as he knows, the first ones shown by the X-ray. The kyphos was well marked at the 10th dorsal vertebra and there was a knotted appearance at the point of abscess. There was a long history of spinal trouble. He also showed skiagrams differentiating Osteo Myelitis from Syphilitic Periostitis in the leg. The conditions were well marked and at once gave information that otherwise could be obtained with certainty only after the operations had been commenced and the foci actually reached.

Dr. W. F. Wells read a paper "When and Where Bacterial Vaccines Should Be Used."

Dr. E. C. Thrash said that we are inclined to become empiric in the use of serums and vaccines because of the stock preparations. Immunity and the other effects of this line of treatment require the observation of skilled and trained men in order to produce results that are good and to prevent dangerous conditions from occurring. It would be folly, for instance, to give more poison to a case of acute erysipelas as is sometimes advised. The principal of immunity demands the presence of the antibodies either before the disease makes its attack or during the intermission of the fight when the patient needs some further stimulus after the cells have had a chance to rest. Typhoid carriers show what is a truce between the cells and the bacteria and the same is true of the Diptheria carriers. The cells can be whipped up with antibodies and cure may follow in this line. Tuberculin has been bitterly assailed because it has not been well understood. The time for treatment is to be learned from the condition of the cells and it should be given just when the cells show they are ready for that kind of assistance. Otherwise the tuberculin is in the way. There should be enough resistance to keep on living and to perpetuate the fight until the cells can win and this the tuberculin will furnish if given under the correct indications. There are surely times when antigens may not be given at all.

Dr. A. H. Bunce said that antigens should be given with reference to selected cases and the stage of invasion of the disease in each of these.

In localized infections such as acne, they can be used with marked success, but in the broader cases, one should always consider the matter debatable. We fail sometimes because of the difference in the strains of the organisms. We may have the correct bacteria to make our injections, but the particular strain is wrong and the results are variable on that account. Thus autogenous vaccines when they can be made are efficacious and they alone will be certain to give the correct strain.

Dr. H. M. Lokey said that his experience with the vaccines had been entirely clinical and that he used them successfully in many cases of middle ear trouble and in peritonsilitis. Autogenous vaccines are of undoubted value, but in his line of work it is always difficult to secure the etiologic germ from which to make the vaccine. He related a case of Vincent's Angina, in which as a last resort he had Dr. Gould make an antogenous vaccine. He was surprised to see her make a rapid recovery after this assistance had been given.

Dr. M. T. Davis said he believed the time has passed when serum therapy is limited to the laboratory worker. The general characteristics of the requirements can be readily learned and as a matter of fact, all vaccines are "mixed." Thus they can be as well used so far as the stock is concerned by one man as another. Autogenous vaccine should be used when it is obtainable but valuable time should not be lost waiting for it.

Dr. L. C. Roughlin said that the name—phylacogen—is but a trade name and should not be used in classifying the serums in serum therapy. No one knows exactly what it is. The whole matter is still in an experimental stage and should not be used until a sounder basis has been established. A

polyvalent vaccine is like the old shot gun prescription and may do as much or more harm than good. It certainly is not scientific. The strain of vaccine is not of so much consequence as the individual susceptibility of the patient and this the observant doctor can know.

Dr. S. P. Harris said that we must make a study of immunity before and after disease. He would have liked to hear the phagocytic role discussed more at length in its influence upon disease. Tuberculin has been used more than all the others. It is a stock vaccine. They all contain the same proteid quality and are fundamentally alike in this regard.

Dr. W. F. Shallemberger said that the antimeningicoccic vaccine has been of use in chronic gonorrheal infection in several cases of his lately. Pelvic and knee rheumatism in an adult, gonnorrhoeal vaginitis in a seven year old girl, specific infection when the pelvic organs had been removed and a case of general pyelitis of Dr. Boyd's all improved and recovered after a few doses of this serum. There was practically no reaction in any of them.

Dr. Wells closed and said that serums were used when there was passive immunity and it needed stirring into active states. As to the trade names, he would leave them all out and use "antigens." The question of Immunity was too large for this discussion and was all set out so far as known at present in the text books.

Dr. R. R. Kime read a paper, "The Present Status of Gall-Bladder Surgery.

Dr. G. P. Huguley said that Cholecystitis is a surgical disease sooner or later and that temporary cures by other methods only prolong the trouble. Pellagra often involves the gall bladder and he would add that to the causes Kime had enumerated. Symptoms do not always relate to the gall bladder. Vomiting at night, pyloric spasm and other gastric symptoms are frequently present and are sometimes the only ones. Recently in an operation he had seen a gall stone in the process of formation. It was a soft putty like mass with sufficient density to prevent its breaking down. To contrast this he said that he had operated upon a man of 42 who never had been sick in his life until he had sudden hyperacidity and colic. He removed 84 stones.

Dr. Kime closing said that he recognized pellagra as a cause of this disease. He added that it does not seem as if cases of acute cholecystitis should clear up under the care of the internist.

### Regular Meeting August 20, 1914.

Dr. J. L. Campbell exhibited a specimen of a gangrenous appendix he had removed from behind the caecum 36 hours after the first symptoms appeared. It might have been overlooked in the search. He removed it and left drainage through the flank as well as the initial wound. He had another case the next day and Dr. W. E. Parsons had one the same day. In all three cases of post caecal appendix in two days. Each had double drainage and all did well. He exhibited another specimen which in his opinion is a calcareous foetus that he found in the broad ligament of a negro woman of 68.

Dr. Selman said he had seen something like this in a case in the dissecting room.

Dr. J. O. Kinard read a paper "Post-operative Abdominal Adhesions.

Dr. J. L. Campbell said that this was a subject of grave interest to all surgeons doing abdominal work. He had had several cases including volvulus, Meckles Diverticlum, illeus and the like. Cysts near the ovaries were also a complication. He says he doesn't know how to account for the adhesions and that he is about to use liquid parafin in some experiments on dogs.

Dr. E. C. Cartlege said that the "'Jackson membrane" was probably a matter of infection. The chief seat of adhesions is near the head of the colon and there may be leakage of infection at that point.

Dr. S. T. Barnett said that the omentum is a great preventer of adhesions as a rule and that he would deprecate extensive operations upon it at any time unless they were surely indicated. Rough technic especially the packing off with gauze was the chief cause of adhesions. Eserine Gr. 1-100 will cause high pulse and should be used cautiously.

Dr. Kinard said in closing that he had experienced no heart trouble in the use of eserine and it did increase peristalsis. In the particular case he described, the omentum was long and inflamed and its removal was indicated.

